# Spatial distribution and factors associated with high completed fertility among women aged 40–49 years in Ghana: evidence from the 2022 Ghana Demographic Health Survey

**DOI:** 10.1186/s12978-024-01845-7

**Published:** 2024-07-11

**Authors:** Augustus Osborne, Camilla Bangura, Richard Gyan Aboagye, Florence Gyembuzie Wongnaah, Abdul-Aziz Seidu, Bright Opoku Ahinkorah

**Affiliations:** 1 Department of Biological Sciences, School of Basic Sciences, Njala University, PMB, Freetown, Sierra Leone; 2 School of Population Health, University of New South Wales, Sydney, NSW 2052 Australia; 3 Department of Family and Community Health, Fred N. Binka School of Public Health, University of Health, and Allied Sciences, Hohoe, Ghana; 4 Department of Global Public Health, Karolinska Institutet, Stockholm, Sweden; 5 Centre for Gender and Advocacy, Takoradi Technical University, P.O. Box 256, Takoradi, Ghana; 6 College of Public Health, Medical and Veterinary Sciences, James Cook University, Townsville, QLD 4811 Australia; 7 REMS Consultancy Services Limited, Western Region, Sekondi-Takoradi, Ghana; 8 School of Clinical Medicine, University of New South Wales Sydney, Sydney, Australia

**Keywords:** High fertility women, Ghana, Demographic and health survey

## Abstract

**Background:**

High completed fertility among married and cohabiting women has profound consequences, including straining resources, increasing healthcare challenges, and contributing to educational and gender inequalities. This study examined the factors associated with high completed fertility among married and cohabiting women aged 40–49 years in Ghana.

**Methods:**

Data for the study was sourced from the 2022 Ghana Demographic and Health Survey (GDHS). A spatial map was used to present the women's geographic variations in high completed fertility. A mixed-effect multilevel binary logistic regression analysis was performed to identify the factors associated with high completed fertility. The findings were presented as adjusted odds ratios (aOR) with a 95% confidence interval (CI).

**Results:**

The national proportion of high completed fertility among married and cohabiting women aged 40–49 years in Ghana was 52.0% [48.8, 55.2]. Women who were Ga/Dangme/Ewe by tribe [aOR = 2.32, 95% CI = 1.06, 5.08] had higher odds of high completed fertility than Akans. Women who indicated 6 + as their ideal number of children had a higher [aOR = 5.60, 95% CI = 2.90, 10.82] likelihood of high completed fertility compared to those whose ideal number of children was 0–3. Those who were using contraceptives at the time of the survey had a higher [aOR = 2.31, 95% CI = 1.17, 4.55] likelihood of high completed fertility compared to those who were not using contraceptives. Women with secondary/higher education [aOR = 0.32, 95% CI = 0.17, 0.58] had lower odds of high completed fertility than those without no formal education. Women with females as household heads [aOR = 0.56, 95% CI = 0.33, 0.95] had lower odds of high completed fertility than males. Women in Volta, Western North, Ahafo, and Bono regions had lower odds of high completed fertility compared to those living in the Northeast region, with the lowest odds among those living in the Volta region [aOR = 0.08, 95% CI = 0.02, 0.40].

**Conclusion:**

High completed fertility is prevalent in Ghana, with more than half of married and cohabiting women having at least five or more children. The government and policymakers in Ghana should promote education for women, increase culturally sensitive family planning programs, increase access to family planning resources, address ideal family size preferences, and improve understanding of contraceptive use.

**Supplementary Information:**

The online version contains supplementary material available at 10.1186/s12978-024-01845-7.

## Introduction

High fertility (five or more children) plays a crucial role in determining reproductive trends, and it is impacted by various interconnected factors such as age, marital status, income, educational level, and parity [1–3]. Factors such as socio-economic status, education, maternal age, number of children ever born, infant mortality, and the opinions of influential individuals regarding desired family size have been found to have a substantial impact on fertility choices [4–7].

Women who have had five or more pregnancies have a higher likelihood of experiencing maternal death [8–11]. Furthermore, countries with high completed fertility rates exhibit low child survival rates [12]. For example, in African countries with high fertility rates, there is a maternal mortality rate of 640 deaths per 100,000 births during pregnancy and childbirth [13]. Population growth in SSA is surpassing economic growth [14]. Many countries have fertility rates that exceed replacement levels [15]. At a replacement fertility level of 2.1, African mothers give birth to almost two daughters, resulting in a significant increase in population [14].

In Ghana, the total fertility rate has experienced a gradual decrease from 6.4 children per woman in 1988 to 4.2 children per woman in 2014 [16], resulting in a reduction of around two births per woman over twenty-six years. Moreover, the 2017 Ghana Maternal Health Survey data indicates that the fertility rate has fallen further to 3.9 [17]. Ghana's 1994 National Population Policy aimed to decrease the fertility rate from 5.5 to 3.0 by 2020 [18]. Given the declining rate, it is not unexpected that the National Population Policy's goal of achieving a total fertility rate of 3.0 per woman by 2020 was unmet [18].

Although there have been studies on childhood mortality [19, 20], intra-household bargaining power [19], and fertility preferences among women in Ghana [21–23], there is limited data on the factors that contribute to high total fertility rates among married and cohabiting women aged 40–49 in Ghana. This age group presents a unique opportunity for intervention, as these women have already completed a significant portion of their childbearing years but may still desire or have the capacity for additional children. This study examines the predictors of high total completed fertility among married and cohabiting women aged 40–49 years in Ghana by analysing data from the 2022 Ghana Demographic and Health Survey (GDHS). Examining these factors can shed light on the complex decision-making processes surrounding family size and inform targeted interventions to promote informed reproductive choices.

## Methods

### Data source

This study employed the 2022 GDHS, a component of the international Demographic and Health Survey (DHS) program that gathers health and demographic information on women, men, and children worldwide [24]. The DHS has been conducted in over 90 LMICs since its establishment, with more than 350 surveys [25]. The 2022 DHS in Ghana is the eighth standard DHS conducted since the first survey in 1988 [24]. The data collection process utilised structured questionnaires, employing a cross-sectional design and a multistage sample procedure [26]. The study comprised 2231 married and cohabiting women aged 40 to 49 years in Ghana. This study followed the Strengthening the Reporting of Observational Studies in Epidemiology (STROBE) checklist [27].

### Variables

High completed fertility was the outcome variable, defined in the DHS as the total number of children ever born by women. We recoded the variable into low and high total fertility by assigning a value of 1 to individuals with five or more children (high) and a value of 0 to individuals who had four or fewer children (low) based on the 2022 GDHS report [24].

### Explanatory variables

Following a detailed literature review on predictors of high completed fertility [28, 29], we included nineteen explanatory variables based on their availability in the GDHS. The variables consisted of the current working status(working, not working), educational level(No education, primary, secondary/higher), marital status(married, cohabiting), listening to the radio(yes, no), watching television (yes, no), reading newspapers or magazines(yes, no), internet use(yes, no), ethnicity(Akan, Ga/Dangme/Ewe, Mole dagbani, Others), religion(Christian, Islam Traditional/no religion), partner educational level(No education, primary, secondary/higher), the ideal number of children(0–3, 4–5, 6 +), decision-making on healthcare(respondent alone, Joint with partner, others), contraceptive use(yes, no), wealth index(poorest, poorer, middle, richer, richest), sex of household head(female, male), type of place of residence(rural, urban), and region(Western, Central, Greater Accra, Volta, Eastern, Ashanti, Western North, Ahafo, Bono, Bono East, Oti, Northern, Savannah, Northeast, Upper East, Upper West). Further, the variables were segregated into individual and contextual levels. Aside from the latter four variables, grouped as contextual (household and community level variables), the remaining were individual-level variables. Please see the attached Supplementary file that shows the coding scheme of the variables.

### Statistical analyses

The statistical analyses were conducted using Stata software version 17.0 (Stata Corporation, College Station, TX, USA). First, a spatial map was used to show the proportion of women with high total fertility. Next, we determined the distribution of the explanatory variables across the outcome variable and used a Pearson chi-square test to show their associations. Finally, a mixed-effect multilevel binary logistic regression analysis was conducted using four models to identify the high completed fertility predictors. Model I, which did not include any explanatory variables, revealed the changes in high completed fertility ascribed to the clustering at the primary sampling units (PSU). In Model II, the individual level variables were included, while in Model III, the contextual level variables were included. Model IV included all the explanatory variables. The mixed-effect regression analysis yielded results that included both fixed effects and random effects. The fixed-effect analysis revealed the correlation between the explanatory predictors and high completed fertility. The results were reported as an adjusted odds ratio (aOR) and their corresponding 95% confidence intervals (CI). The random effect results, however, indicate the variations in high completed fertility. All four models used the intra-cluster correlation coefficient (ICC) values to determine the variation. All the analyses were weighted, and the svyset command in Stata, which contains the sampling weights, one or more stages of clustered sampling, and stratification, was used to deal with the complex nature of the DHS dataset.

### Ethical consideration

Since the GDHS dataset is publicly available, we did not require a separate ethical clearance process for this study. However, we ensured proper access by obtaining permission to use the data for publication from MEASURE DHS (Monitoring and Evaluation to Assess and Use Results—Demographic and Health Surveys). For more information on ethical considerations and privacy principles related to using DHS data, please refer to the MEASURE DHS website: https://dhsprogram.com/Methodology/Protecting-the-Privacy-of-DHS-Survey-Respondents.cfm.

## Results

### Background characteristics of the married and cohabiting women in Ghana

Table 1 shows the background characteristics of married and cohabiting women in Ghana. Nearly half of the women (49.1%) had a secondary or higher education. Most (83.4%) were married, and a significant portion (73.9%) identified as Christian. Interestingly, a high percentage (92.1%) were employed, yet most (72.4%) had never used the internet. Looking at ethnicity, 46.1% belonged to the Akan tribe. Finally, the wealth distribution showed that 22.2% were richer and a higher proportion (53.2%) living in urban areas.

**Table 1:** Background characteristics of married and cohabiting women aged 40–49 years in Ghana

Variable	Weighted sample(n)	Weighted frequency(%)
**Educational attainment**		
No education	750	33.6
Primary	385	17.3
Secondary/higher	1,096	49.1
**Marital status**		
Married	1,862	83.4
Cohabiting	369	16.6
**Religion**		
Christians	1,648	73.9
Muslims	461	20.7
Traditional/no religion	122	5.4
**Current working status**		
Not working	177	7.9
Working	2,054	92.1
**Read newspapers or magazines**		
No	2,089	93.6
Yes	142	6.4
**Listen to radio**		
No	627	28.1
Yes	1,604	71.9
**Watch television**		
No	624	28.0
Yes	1,607	72.0
**Use Internet**		
No	1,615	72.4
Yes	616	27.6
**Ethnicity**		
Akan	1,028	46.1
Ga/Dangme/Ewe	380	17.1
Mole dagbani	421	18.9
Others	401	17.9
**Partners educational attainment**		
No education	659	29.5
Primary	177	8.0
Secondary/higher	1,395	62.5
**Ideal number of children**		
0–3	425	19.1
4–5	885	39.7
6 +	921	41.3
**Decision on healthcare**		
Respondent	1,015	45.5
Joint	768	34.4
Others	448	20.1
**Contraceptive use**		
No	1,529	68.5
Yes	702	31.5
**Sex of household head**		
Male	1,652	74.0
Female	579	26.0
**Wealth index**		
Poorest	430	19.3
Poorer	432	19.4
Middle	406	18.2
Richer	466	20.9
Richest	497	22.2
**Place of residence**		
Urban	1,187	53.2
Rural	1,044	46.8
**Region**		
Western	126	5.6
Central	235	10.5
Greater Accra	312	14.0
Volta	116	5.22
Eastern	204	9.1
Ashanti	428	19.1
Western North	66	2.97
Ahafo	55	2.4
Bono	83	3.7
Bono East	93	4.1
Oti	67	3.0
Northern	189	8.4
Savannah	53	2.42
Northeast	44	1.9
Upper East	93	4.2
Upper West	65	2.9

### Proportion of high completed fertility among married and cohabiting women aged 40 – 49 years in Ghana

**Fig. 1:**
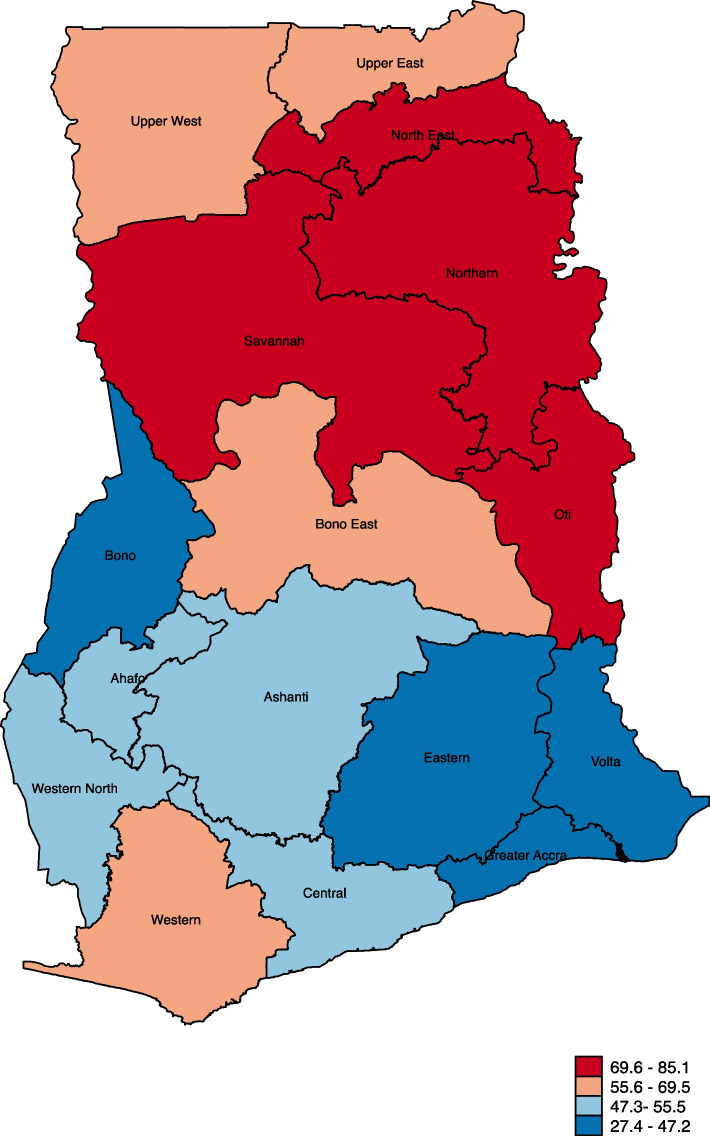
Proportion of high completed fertility among married and cohabiting women aged 40 – 49 years in Ghana

Figure 1 presents the proportion of high completed fertility among married and cohabiting women aged 40–49 years in Ghana. High completed fertility was high among married and cohabiting women living in the Northeast 85.1%, Oti 78.7%, Savannah 74.5%, and Northern region 71.6% whilst married and cohabiting women in the Bono 45.2%, Eastern 44.8%, Volta 39.3%, and Greater Accra regions 27.4% had the lowest proportion of high completed fertility in Ghana. The national proportion of high completed fertility among married and cohabiting women aged 40 – 49 years in Ghana was 52.0 [48.8, 55.2] (Table 3).

### Bivariate results of the association between the explanatory variable and high completed fertility

Table 2 presents the bivariable analysis of the proportion and distribution of high completed fertility among married and cohabiting women aged 40 – 49 years in Ghana. High completed fertility was high among women who had no education 74.0%, who were Mole Dagbani by tribe 66.0%, whose partner had no education 73.3%, whose ideal number of children 72.5% was 6 + , who used contraceptives 63.3%, who were poorest 78.8%, who lived in rural areas 52.0% and women who live in the Northeast region 85.1%. Marital status was the explanatory variable not significantly associated with high completed fertility among married and cohabiting women aged 40 – 49 years in Ghana at *p* < 0.05.

**Table 2:** Bivariable analysis of high completed fertility among married and cohabiting women aged 40 – 49 years in Ghana

Variables	High completed fertility	*p*-value
**Proportion**	**52.0% [48.8, 55.2]**	
**Educational attainment**		< 0.001
No education	74.0 [70.0, 77.6]	
Primary	59.9 [53.8, 65.8]	
Secondary/higher	34.2 [29.9, 38.8]	
**Marital status**		0.598
Married	52.4 [48.9,55.8]	
Cohabiting	50.3 [43.2,57.4]	
**Religion**		< 0.001
Christians	46.9 [43.3, 50.6]	
Muslims	65.4 [60.4, 70.1]	
Traditional/ no religion	70.6 [57.8, 80.8]	
**Current working status**		0.015
Not working	41.7 [33.1, 50.9]	
Working	52.9 [49.7,56.1]	
**Read newspapers or magazines**		< 0.001
No	54.7 [51.5, 57.9]	
Yes	12.6 [6.7, 22.4]	
**Listen to radio**		< 0.001
No	60.2 [55.2, 65.0]	
Yes	48.8 [45.2, 52.5]	
**Watch television**		< 0.001
No	69.8 [65.1, 74.1]	
Yes	45.1 [41.3, 49.0]	
**Use Internet**		< 0.001
No	61.6 [58.2, 64.8]	
Yes	27.0 [21.6, 33.3]	
**Ethnicity**		< 0.001
Akan	43.7 [39.1, 48.3]	
Ga/Dangme/Ewe	44.5 [37.7,51.6]	
Mole Dagbani	66.0 [61.1, 70.6]	
Others	65.9 [59.1, 72.2]	
**Partners educational attainment**		< 0.001
No education	73.3 [68.9, 77.3]	
Primary	67.9 [59.5, 75.3]	
Secondary/higher	40.0 [36.1, 44.1]	
**Ideal number of children**		< 0.001
0–3	32.9 [27.2, 39.2]	
4–5	40.0 [35.5, 44.7]	
6 +	72.5 [68.5, 76.1]	
**Decision on healthcare**		< 0.001
Respondent	47.5 [42.7, 52.2]	
Joint	52.7 [47.7, 57.8]	
Others	61.2 [55.4, 66.7]	
**Contraceptive use**		< 0.001
No	46.9 [43.0, 50.8]	
Yes	63.3 [58.2, 68.1]	
**Sex of household head**		< 0.001
Male	55.4 [51.6, 59.2]	
Female	42.4 [37.0, 48.0]	
**Wealth index**		< 0.001
Poorest	78.8 [74.3, 82.7]	
Poorer	69.0 [64.0, 73.6]	
Middle	58.0 [51.0, 64.7]	
Richer	34.3 [28.1, 41.0]	
Richest	26.0 [19.5, 33.7]	
**Place of residence**		< 0.001
Urban	38.4 [33.9, 43.1]	
Rural	52.0 [48.8, 55.2]	
**Region**		< 0.001
Western	56.1 [44.4, 67.1]	
Central	55.1 [44.7, 65.1]	
Greater Accra	27.4 [18.6, 38.3]	
Volta	39.3 [30.4, 49.0]	
Eastern	44.8 [37.3, 52.5]	
Ashanti	49.4 [40.8, 58.1]	
Western North	50.4 [40.9, 59.8]	
Ahafo	54.7 [48.2, 61.1]	
Bono	45.2 [35.6, 55.2]	
Bono East	61.9 [49.3, 73.0]	
Oti	78.7 [69.7, 85.5]	
Northern	71.6 [62.7, 79.1]	
Savannah	74.5 [65.9, 81.5]	
Northeast	85.1 [75.4, 91.4]	
Upper East	62.9 [53.5, 71.5]	
Upper West	67.5 [59.0, 74.9]	

^*^P-values were generated from a Chi-square test

### Factors associated with high completed fertility among married and cohabiting women aged 40–49 years in Ghana

#### Fixed effect results

Model IV of Table 3 which include the individual level and contextual level variables show the factors associated with high completed fertility among married and cohabiting women aged 40–49 years in Ghana. Women with secondary/higher education [aOR = 0.32, 95% CI = 0.17, 0.58] had lower odds of high completed fertility than those without no formal education. Women who read newspapers or magazines [aOR = 0.09, 95% CI = 0.02, 0.40] had lower odds of high completed fertility than those who didn't. Women with females as household heads [aOR = 0.56, 95% CI = 0.33, 0.95] had lower odds of high completed fertility than males. Women in Volta, Western North, Ahafo, and Bono regions had lower odds of high completed fertility compared to those living in the Northeast region, with the lowest odds among those living in the Volta region [aOR = 0.08, 95% CI = 0.02, 0.40]. Women who were Ga/Dangme/Ewe by tribe [aOR = 2.32, 95% CI = 1.06, 5.08] had higher odds of high completed fertility than Akans. Women who indicated 6 + as their ideal number of children had a higher [aOR = 5.60, 95% CI = 2.90, 10.82] likelihood of high completed fertility compared to those whose ideal number of children was 0–3. Those who were using contraceptives at the time of the survey had a higher [aOR = 2.31, 95% CI = 1.17, 4.55] likelihood of high completed fertility than those who were not using contraceptives.

**Table 3:** Factors associated with high completed fertility among married and cohabiting women aged 40–49 years in Ghana

Variables	Model I Empty model	Model II aOR [95% CI]	Model III aOR [95% CI]	Model IV aOR [95% CI]
**Fixed effect results**				
** Educational attainment**				
No education		1.00		1.00
Primary		0.60 [0.33,1.09]		0.66 [0.36,1.21]
Secondary/higher		0.26^***^ [0.15,0.47]		0.32^***^ [0.17,0.58]
** Religion**				
Christians		1.00		1.00
Muslims		1.54 [0.77,3.10]		1.46 [0.73,2.95]
Traditional/no religion		1.66 [0.61,4.54]		1.72 [0.66,4.46]
** Current working status**				
Not working		1.00		1.00
Working		1.63 [0.79,3.36]		1.56 [0.75,3.24]
** Read newspaper or magazine**				
No		1.00		1.00
Yes		0.08^**^ [0.02,0.37]		0.09^**^ [0.02,0.40]
** Listen to radio**				
No		1.00		1.00
Yes		0.88 [0.54,1.44]		0.90 [0.54,1.50]
** Watch television**				
No		1.00		1.00
Yes		0.84 [0.48,1.47]		1.03 [0.59,1.80]
** Use Internet**				
No		1.00		1.00
Yes		0.48^*^ [0.27,0.87]		0.63 [0.33,1.19]
** Ethnicity**				
Akan		1.00		1.00
Ga/Dangme/Ewe		2.13^*^ [1.03,4.38]		2.32^*^ [1.06,5.08]
Mole Dagbani		0.67 [0.26,1.74]		0.73 [0.26,2.03]
Others		1.48 [0.64,3.40]		1.61 [0.67,3.85]
** Partner educational attainment**				
No education		1.00		1.00
Primary		0.91 [0.42,1.99]		0.87 [0.40,1.91]
Secondary/higher		0.93 [0.47,1.84]		1.01 [0.49,2.05]
**Ideal number of children**				
0–3		1.00		1.00
4–5		1.31 [0.71,2.41]		1.31 [0.72,2.39]
6 +		5.58^***^ [2.92,10.68]		5.60^***^ [2.90,10.82]
** Decision on healthcare**				
Respondent		1.00		1.00
Joint		1.62 [0.98,2.69]		1.71^*^ [1.03,2.83]
Others		1.63 [0.88,3.04]		1.57 [0.83,2.95]
** Contraceptive use**				
No		1.00		1.00
Yes		5.09^***^ [2.93,8.84]		2.31^*^ [1.17,4.55]
** Sex of household head**				
Male			1.00	1.00
Female			0.59^*^ [0.35,0.98]	0.56^*^ [0.33,0.95]
** Wealth index**				
Poorest			1.00	1.00
Poorer			0.98 [0.50,1.90]	1.26 [0.60,2.61]
Middle			0.93 [0.42,2.06]	1.26 [0.51,3.07]
Richer			0.35^*^ [0.14,0.85]	0.51 [0.18,1.43]
Richest			0.17^***^ [0.07,0.45]	0.37 [0.12,1.13]
** Place of residence**				
Urban			1.00	1.00
Rural			4.39^***^ [2.42,7.94]	3.71^***^ [1.93,7.13]
** Region**				
Western			0.50 [0.14,1.80]	1.41 [0.32,6.17]
Central			0.23^*^ [0.07,0.78]	0.72 [0.16,3.16]
Greater Accra			0.12^**^ [0.03,0.48]	0.32 [0.06,1.63]
Volta			0.06^***^ [0.02,0.20]	0.08^**^ [0.02,0.40]
Eastern			0.13^***^ [0.04,0.42]	0.26 [0.06,1.09]
Ashanti			0.28^*^ [0.09,0.90]	0.75 [0.20,2.83]
Western North			0.07^***^ [0.02,0.26]	0.15^*^ [0.03,0.67]
Ahafo			0.12^***^ [0.04,0.35]	0.19^*^ [0.05,0.70]
Bono			0.13^**^ [0.04,0.47]	0.25^*^ [0.06,0.99]
Bono East			0.21^*^ [0.06,0.69]	0.52 [0.14,1.95]
Oti			1.29 [0.36,4.65]	1.74 [0.39,7.84]
Northern			0.50 [0.16,1.54]	0.50 [0.15,1.63]
Savannah			0.48 [0.14,1.65]	0.32 [0.09,1.21]
Northeast			1.00	1.00
Upper East			0.16^**^ [0.04,0.56]	0.30 [0.08,1.12]
Upper West			0.26^*^ [0.08,0.84]	0.38 [0.11,1.32]
** Random effect model**				
PSU variance (95% CI)	8.35 [6.03, 11.56]	8.51 [5.61, 12.91]	5.03 [3.68, 6.83]	8.17 [5.46, 12.21]
ICC	0.72 [0.65, 0.78]	0.72 [0.63, 0.80]	0.60 [0.53,0.68]	0.71 [0.62,0.79]
N	2231	2231	2231	2231
Number of clusters	597	597	597	597

*aOR* Adjusted odds ratios, *CI* Confidence Interval; * *p* < ; 0.05, ** *p* < ; 0.01, *** *p* < ; 0.001; 1.00 = Reference category, *PSU* Primary Sampling Unit, *ICC* Intra-Class Correlation Coefficient

#### Random effect results

Table 3 indicates considerable variations in the predictors associated with high completed fertility among married and cohabiting women in Ghana among the clusters (σ2 = 8.35, 95% CI = 6.03 to 11.56) in the model I. Approximately 72% of the proportion of high completed fertility was attributed to the variations between the clusters (intra-cluster correlation coefficient (ICC) = 0.72). The between-cluster difference was maintained at 72% in Model II, decreased to 60% in Model III, and increased to 71% in Model IV. These ICC results suggest that the likelihood of high completed fertility variations can be attributed to the variances across the clusters.

## Discussion

Our study examines the predictors of high completed fertility among married and cohabiting women aged 40–49 in Ghana. Over half (52.0%) of married and cohabiting women aged 40–49 years in Ghana have a high completed fertility rate. Education, ethnicity, reading newspapers or magazines, ideal number of children, contraceptive use, healthcare decisions, sex of household head, residence, and region of residence were all associated with high completed fertility among married and cohabiting women aged 40 – 49 years in Ghana.

The study found that 52.0% of women aged 40–49 in Ghana have a high completed fertility. Both place of residence and region were found to be associated with high completed fertility, with women living in rural areas having higher odds of higher completed fertility than those living in urban areas. Rural areas in Ghana often have limited access to healthcare facilities and family planning services. This can make it difficult for women to obtain contraception or get proper counselling on reproductive health options [30]. Educational attainment tends to be lower in rural areas compared to urban centres. Educated women in Ghana are more likely to have smaller families and make informed decisions about family planning [31]. Cultural norms in some rural communities might emphasise the importance of large families. Having many children can be seen as a sign of prosperity, social status, and security in old age [32]. With region, women in Volta, Western North, Ahafo, and Bono had lower odds of high completed fertility than those in Ghana's Northeast region. Volta, Western North, Ahafo, and areas of Bono might have a higher average education level for women compared to the Northeast region [33].

Women with secondary and higher education have lower odds of a high completed fertility rate than those with no education in Ghana. Our finding is consistent with the previous study in Nigeria [34]. Education can teach women about family planning methods, effectiveness, and accessibility [35]. Education can empower women to make informed choices about their reproductive health and family size [35]. One of the avenues through which education impacts fertility levels is exposure to media. This affirms our finding that women who read newspapers or magazines have lower odds of higher completed fertility compared to those who don't read newspapers or magazines. Newspapers and magazines can provide information on family planning methods and reproductive health to educated women who read them [36]. By reading these publications, women may learn about different contraceptive options, their effectiveness, and where to access them. This increased knowledge could empower them to make informed choices about family planning.

Moreover, we found ethnicity to be associated with high completed fertility, with women who are Ga/Dangme/Ewe by tribe having higher odds of a high completed fertility rate compared to those who are Akan by tribe in Ghana. Lower average education levels within the Mande ethnic group could lead to less awareness or acceptance of family planning methods [37]. Societal expectations and cultural and religious beliefs about family size and gender roles can influence fertility decisions [38]. In some communities, having many children may be valued or expected. Economic instability and poverty can also lead to higher fertility rates as families may perceive children as a source of labour or financial security in old age if that still exists in certain tribes and communities. However, the wealth index was not significant.

Also, decision-making power was significantly associated with high completed fertility. The study found that women whose decision on healthcare is made by both respondent and partner (Joint) have higher odds of higher completed fertility than those whose decision on healthcare is made by respondent alone. Further research would be needed to explain the possible reasons for the association. We also found that women with females as household heads have lower odds of higher completed fertility than males. Women in female-headed households might have social networks that are more supportive of family planning. They could be exposed to information and experiences of other women who have chosen smaller families [39]. In female-headed households, women have more autonomy in making decisions about their reproductive health. They might feel more empowered to use contraception without needing a partner's approval [39].

### Policy and practice implications

The study found a strong link between education and lower fertility rates. Policies that increase access to and completion rates of secondary education for girls could be impactful. The association between a higher ideal family size and higher completed l fertility suggests a need for family planning services that address the desired family size. This could involve counselling, education about different family planning methods, and ensuring the affordability of these methods. The finding that contraceptive use is associated with higher completed fertility needs further exploration. Research into why this might be the case could inform improvements in family planning programs. This could involve offering a more comprehensive range of contraceptive methods or promoting more consistent use. The study identifies ethnicity as a factor influencing fertility rates. Family planning programs should be designed to be sensitive to the cultural values and beliefs of different ethnic groups. The study shows regional variations in fertility rates. Policies and programs promoting family planning could be targeted to regions with higher fertility rates. Policies promoting shared decision-making between partners regarding family planning can be encouraged. This could involve educational campaigns highlighting the benefits of joint discussions about family size and contraceptive methods. The government and policymakers in Ghana should invest in creating male-focused reproductive health services and educational programs. This could involve designated clinic hours for men, offering male-specific counselling on family planning options, and addressing social stigmas surrounding male involvement in reproductive health.

### Strengths and limitations

One of the study's strengths is that it uses the 2022 GDHS, a nationally representative sample, allowing for generalisable conclusions about the population of interest (married and cohabiting women aged 40–49). The GDHS collects data on various demographic, social, and economic factors. This allows for exploring the complex relationships between these factors and fertility rates. Lastly, the 2022 data provides the most recent information on fertility trends in Ghana. This study, however, has some limitations. The DHS is a cross-sectional survey, which limits the ability to establish causal relationships between factors and fertility [40]. Fertility data relies on women's self-reports, which can be subject to recall or social desirability bias.

## Conclusion

Over half of married and cohabiting women aged 40–49 years in Ghana have a high completed fertility. Education, ethnicity, reading newspapers or magazines, the ideal number of children, contraceptive use, healthcare decisions, sex of household head, residence, and region of residence were all associated with high completed fertility among married and cohabiting women in Ghana. The government and policymakers in Ghana should promote education for women, increase culturally sensitive family planning programs, increase access to family planning resources, address ideal family size preferences, improve understanding of contraceptive use, and strengthen health systems, particularly at the primary healthcare level, is essential for improving access to family planning, and other reproductive healthcare services.

### Supplementary Information

Supplementary Material 1.

## Data Availability

The data used for this study is freely available at http://dhsprogram.com/data/available-datasets.cfm.

## Abbreviations

aOR: Adjusted Odds Ratio
CI: Confidence Interval
DHS: Demographic and Health Survey
GDHS: Ghana Demographic and Health Survey
ICC: Intra-Class Correlation Coefficient
MEASURE DHS: Monitoring and Evaluation to Assess and Use Results Demographic and Health Surveys
PSU: Primary Sampling Unit
SSA: Sub-Saharan Africa
STROBE: Strengthening the Reporting of Observational Studies in Epidemiology
