# Supplementary material for: Spatial distribution and factors associated with high completed fertility among women aged 40–49 years in Ghana: evidence from the 2022 Ghana Demographic Health Survey
Source: Reprod Health. 2024 Jul 11;21:104. doi: 10.1186/s12978-024-01845-7 (PMC11238405; doi:10.1186/s12978-024-01845-7)
Supplement: Supplementary file 1 — Supplementary Material 1. [file 12978_2024_1845_MOESM1_ESM.docx]

Table 1: Explanatory variables coding scheme

| Variables | Survey question | Original response options | Recoded |
| --- | --- | --- | --- |
| Educational attainment | highest educational level | 0=No education  1= Primary  2= Secondary  3= Higher | 0=No education  1= Primary  2/3=Secondary/higher |
| Marital status | current marital status | 0=never in union  1= married  2= living with partner  3= widowed  4= divorced  5= no longer living together/separated | 1= married  2= living with partner |
| Religion | religion | 1. catholic 2. Anglican 3. methodist 4. presbyterian 5. Pentecostal/charismatic 6. other Christian 7. Islam 8. traditional/spiritualist 9. no religion 10. Other | 1/6=Christians  7= Muslim  8/10= Traditional/no religion |
| Current working status | respondent currently working | 0=no  1=yes | 0=not working  1=working |
| Read newspapers or magazines | frequency of reading newspapers or magazine | 0= not at all  1= less than once a week  2= at least once a week | 0=no  1/2=yes |
| Listen to radio | frequency of listening to the radio | 0= not at all  1= less than once a week  2= at least once a week | 0=no  1/2=yes |
| Watch television | frequency of watching television | 0= not at all  1= less than once a week  2= at least once a week | 0=no  1/2=yes |
| Use Internet | frequency of using the internet | 0= not at all  1= less than once a week  2= at least once a week  3=almost everyday | 0=no  1/3=yes |
| Ethnicity | ethnicity | 1= Akan  2= Ga/dangme  3= Ewe  4=Guan  5= Mole-Dagbani  6= Grusi  7=Gurma  8=Mande  9=other | 1= Akan  2/3=Ga/dangme/Ewe  5= Mole-dagbani  4,6/9=Others |
| Partners educational attainment | Partner highest educational level | 0=No education  1= Primary  2= Secondary  3= Higher | 0=No education  1= Primary  2/3=Secondary/higher |
| Ideal number of children | ideal number of children | 0, 1,2,3,4,5, 6+ | 0-3  4-5  6+ |
| Decision on healthcare | person who usually decides on: respondent's health care | 1= respondent alone  2= respondent and husband/partner  4= husband/partner alone  5= someone else  6= Other | 1= respondent alone  2= respondent and husband/partner  4/6= Others |
| Contraceptive use | current contraceptive method | 0 not using  1 pill  2 iud  3 injections  5 male condom  6 female sterilisation  8 periodic abstinence  9 withdrawal  10 other traditional  11 implants/Norplant  13 lactational amenorrhea (lam)  16 emergency contraception  17 other modern method  18 standard days method (sdm | 0=no  1/18=yes |
| Sex of household head | sex of household head | 1=Male  2=Female | 1=Male  2=Female |
| Wealth index | wealth index | 1 poorest  2 poorer  3 middle  4 richer  5 richest | 1 poorest  2 poorer  3 middle  4 richer  5 richest |
| Place of residence | type of place of residence | 1= Urban  2=Rural | 1= Urban  2=Rural |
| Region | region | 1 western  2 central  3 greater accra  4 volta  5 eastern  6 ashanti  7 western north  8 ahafo  9 bono  10 bono east  11 oti  12 northern  13 savannah  14 north east  15 upper east  16 upper west | 1 western  2 central  3 greater accra  4 volta  5 eastern  6 ashanti  7 western north  8 ahafo  9 bono  10 bono east  11 oti  12 northern  13 savannah  14 north east  15 upper east  16 upper west |
